# Avoiding Toxic Levels of Essential Minerals: A Forgotten Factor in Deer Diet Preferences

**DOI:** 10.1371/journal.pone.0115814

**Published:** 2015-01-23

**Authors:** Francisco Ceacero, Tomás Landete-Castillejos, Augusto Olguín, María Miranda, Andrés García, Alberto Martínez, Jorge Cassinello, Valentín Miguel, Laureano Gallego

**Affiliations:** 1 Department of Animal Science and Food Processing, Czech University of Life Sciences, Prague, Czech Republic; 2 Department of Ethology, Institute of Animal Science, Prague, Czech Republic; 3 Sección de Recursos Cinegéticos y Ganaderos, Instituto de Desarrollo Regional (IDR), Albacete, Spain; 4 Instituto de Investigación en Recursos Cinegéticos (IREC), CSIC-UCLM-JCCM, Ciudad Real, Spain; 5 Departamento de Ciencia y Tecnología Agroforestal y Genética, Universidad de Castilla-La Mancha, Albacete, Spain; 6 Colegio de Postgraduados Campus Córdoba, Veracruz, Mexico; 7 Centre for African Ecology, School of Animal, Plant and Environmental Sciences, University of the Witwatersrand, Johannesburg, South Africa; 8 Laboratorio de Ciencia e Ingeniería de Materiales, Instituto de Desarrollo Regional (IDR), Albacete, Spain; University of Bari, ITALY

## Abstract

Ungulates select diets with high energy, protein, and sodium contents. However, it is scarcely known the influence of essential minerals other than Na in diet preferences. Moreover, almost no information is available about the possible influence of toxic levels of essential minerals on avoidance of certain plant species. The aim of this research was to test the relative importance of mineral content of plants in diet selection by red deer (*Cervus elaphus*) in an annual basis. We determined mineral, protein and ash content in 35 common Mediterranean plant species (the most common ones in the study area). These plant species were previously classified as preferred and non-preferred. We found that deer preferred plants with low contents of Ca, Mg, K, P, S, Cu, Sr and Zn. The model obtained was greatly accurate identifying the preferred plant species (91.3% of correct assignments). After a detailed analysis of these minerals (considering deficiencies and toxicity levels both in preferred and non-preferred plants) we suggest that the avoidance of excessive sulphur in diet (i.e., selection for plants with low sulphur content) seems to override the maximization for other nutrients. Low sulphur content seems to be a forgotten factor with certain relevance for explaining diet selection in deer. Recent studies in livestock support this conclusion, which is highlighted here for the first time in diet selection by a wild large herbivore. Our results suggest that future studies should also take into account the toxicity levels of minerals as potential drivers of preferences.

## Introduction

Diet selection by deer at the individual food items level is a highly complex problem because of the enormous nutritional heterogeneity of food resources [1]. A large body of studies testing the Optimal Foraging Theory predictions (OFT [2]) has repeatedly shown that large herbivores aim to maximize the intake of energy [3] or protein [4], while minimizing at the same time the energetic and temporal costs of resource search [5,6], and avoiding toxic chemicals [7–9]. Large herbivores are able to select a balanced mixture of nutrients, which includes the detection of mineral imbalances and the selection for plants or supplements rich in deficient minerals [10–17]. However, the proportion of studies on diet selection that analyze the influence of essential minerals on such selection has been rather scarce during the last century [1,18], in spite of the interest of mineral contents due to their often deficient levels in food resources available to ungulates and the fact that they can easily reach toxicity [19]. Available literature suggests that the mineral seeking behaviour is related to availability and consumers’ requirements, and is highly context-dependent: even though some mineral deficiencies are widespread, they are soil and habitat-specific. However, almost nothing is known about the opposite *i.e.*, studies on avoidance of foods rich in toxic mineral elements are lacking in the scientific literature, especially studies on free ranging populations. Diet selection studies should consider any factor susceptible of causing negative post-ingestive feedbacks, and thus of influencing diet preferences [13], but toxic levels of essential minerals have only very recently started to be considered. In particular, sulphur is a good candidate to study toxicity in ruminant diets, since recent experiments in livestock have highlighted the negative effect of diets with S levels above 0.2% [20–22], confirmed this effect though meta-analysis [23], and the topic has recently been even subject of review [24].

The aim of this study was to determine to what extent plant preferences by Iberian red deer (*Cervus elaphus hispanicus*) in natural habitats are linked to mineral content, considering not only the effect of deficiencies of essential minerals, but also the possible influence of toxic concentrations.

## Materials and Methods

### Ethics Statement

The study was conducted in a private game estate under the permission of the owners and managers Yolfi S.L. (Mrs. Yolanda Fierro). No other permission was needed since the work did not involve the manipulation of animals.

### Study Area

The study was conducted in a 700 ha fenced hunting estate (Abenojar, Ciudad Real, Spain; 30.9°N, 4.2°W, 600–830 m.a.s.l.). The climate is Mediterranean, soil is calcareous and the habitat is characterized by scrubland with evergreen oak (*Quercus ilex*) and rock rose (*Cistus spp*.), with scattered pastures and small areas of crops. European wild boar (*Sus scrofa*), and a few introduced Barbary sheep (*Ammotragus lervia*) and mouflon (*Ovis orientalis musimon*) also inhabit the area. Deer density is estimated at 33 individuals per 100 ha. A selective culling scheme is applied to control population density, reducing herbivory level and damage to the habitat in order to keep the populations in good condition and maximum trophy size. Regular supplementation with pure-salt blocks is an habitual practice, and thus, results related to Na should be treated with caution. Supplementary concentrate (low but unmeasured amounts) is also daily provided in the study site, in order to enhance deer survival and reproduction rates, and promote trophy size and quality [25]. Although this common practice adds artificiality to the study, it allows a better visualization of the plant species selection: it is important for animals to receive an adequate basal diet in order to test preferences under conditions in which nutritional or energetic requirements are satisfied [26].

### Preferred and Rejected Plants by Red Deer

Iberian red deer is classified as intermediate feeder [27,28]. The 35 most abundant plants in the study area and in deer diet were selected to be analyzed. We classified these plants in a binomial way as preferred and non-preferred (Table 1). This selection was based in i) bibliography: studies published for Iberian deer in Mediterranean areas [27–30], ii) 20 years of personal observations in the study area by estate managers and staff, and iii) results from a parallel research on diet selection conducted in the same location [31–33]. Under this criteria, twenty-three plant species were identified as preferred, and 12 as non-preferred. This simultaneous research showed that 27 out of the 35 studied plants (the other 8 were not included in Miranda et al. studies) represented an 80.30% of the biomass available, and added up to 82.25% of the diet of red deer in the study area (measured through microhistological analyses of faecal samples [31–33]).

**Table 1:** List of studied plant species.

**Family**	**Scientific name**	**Category**	**Preference ^a^**
Anacardiaceae	*Pistacia sp*.	Bush/Tree	P *
Boraginaceae	*Heliotropium europaeum*	Herbaceous	NP *
Campanulaceae	*Campanula sp*.	Herbaceous	P *
Cistaceae	*Cistus ladanifer*	Bush/Tree	P *
	*Cistus salviifolius*	Bush/Tree	P *
Asteraceae	*Cirsium tuberosum*	Herbaceous	P
	*Chondrilla juncea*	Herbaceous	P
	*Senecio jacobaea*	Herbaceous	NP
Brassicaceae	*Coronopus didymus*	Herbaceous	NP
	*Raphanus raphanistrum*	Herbaceous	P
Ericaceae	*Arbustus unedo*	Bush/Tree	P *
Fabaceae	*Genista hirsuta*	Bush/Tree	P *
Fagaceae	*Quercus faginea*	Bush/Tree	P *
	*Quercus ilex*	Bush/Tree	P *
	*Quercus coccifera*	Bush/Tree	P *
Gentianaceae	*Centaurium pulchellum*	Herbaceous	P
Globulariaceae	*Globularia allypum*	Bush/Tree	P *
Poaceae	*Elymus repens*	Herbaceous	P *
	*Briza máxima*	Herbaceous	P *
	*Cynodon dactylon*	Herbaceous	P *
Guttiferae	*Hipericum perforatum*	Herbaceous	NP
Labiatae	*Mentha pulegium*	Herbaceous	NP
	*Mentha suaveolens*	Herbaceous	NP
	*Lavandula stoechas*	Bush/Tree	NP *
Leguminosae	*Vicia benghalensis*	Herbaceous	P *
	*Ornithopus compressus*	Herbaceous	P *
	*Trifolium campestre*	Herbaceous	P *
Malvaceae	*Sida abutilon*	Herbaceous	NP *
Oleaceae	*Phillyrea angustifolia*	Bush/Tree	P *
Polygonaceae	*Rumex acetosella*	Herbaceous	NP *
Portulacaceae	*Portulaca oleracea*	Herbaceous	P
Scrophulariaceae	*Verbascum sinuatum*	Herbaceous	NP *
Solanaceae	*Datura stramonium*	Herbaceous	NP
Thymelaeaceae	*Daphne gnidium*	Bush/Tree	NP *
Zygophyllaceae	*Tribulus terrestris*	Herbaceous	P

^a^ P indicates preferred, and NP non-preferred plants. All plants were initially assigned by personal observation by managers and staff of the study site, and thereafter confirmed by bibliography in Mediterranean areas [27–30] and microhistological studies [31–33] (those species marked with *).

### Plant Sampling

Samples of preferred and non-preferred plants were collected in 10 plots randomly distributed in the study area. Collection was carried out seasonally from autumn 2007 to summer 2008 (every 3 months, in the middle of each season). Leaves, stems, and fresh shoots were collected because these are the plant parts consumed by red deer. All the samples collected for each plant species were pooled. Leaves were removed from branches by hand, first air-dried for 2 days in paper bags and later oven-dried at 65°C for 72 h. Thereafter, the material was ground and stored in sterile and hermetic bags. Finally, proportional weights of these 4 seasonal subsamples were pooled in a single sample for chemical and protein analyses. With our annual approach results are not so conclusive, but we have a relatively accurate picture of the potential effects of minerals in diet selection in an annual basis.

### Chemical and Protein Analyses

We analyzed those essential minerals that play an important role in deer physiology (Ca, Mg, P, Na, K, S, B, Cu, Fe, Mn, Se, Sr and Zn [19,34–35]). The analyses were conducted in a sample of 0.5 g (± 0.001g; Gram Model SR-410M, Barcelona, Spain). The samples were dissolved in an acid solution (32% HNO_3_, 12% HCl, 6% HF, and 50% H_2_O). A second wet digestion was carried out in a microwave oven (Perkin-Elmer Multiwave 3000, Boston, USA) below 345 kPa for 30 min. Subsequently, samples were analyzed with an atomic absorption spectrophotometer Optima 5300 DV (Perkin-Elmer ICP-OES, Boston, USA). Results for macro-minerals are shown in percentage, whilst micro-minerals are expressed in parts per million (mg/kg). Ash content was assessed by ashing the samples in a muffle furnace (Carbolite Furnace, Derbyshire, UK) at 550°C for 6 h. Crude protein was determined by the Kjeldahl method on a digester Pro-Nitro M (JP Selecta, Barcelona, Spain) and the valuation was carried out in a 848 Titrino Plus (Metrohm, Switzerland). We did not analyze condensed tannins because deer have tannin binding salivary proteins [36] which reduce the importance of these compounds for diet selection. This has been also observed in our study area, where red deer select plants with high content in tannins [32].

### Statistical Analysis

A one-way ANOVA examined differences in mineral content between preferred and non-preferred plant species (in general and separately for shrubs and herbaceous plants). Correlations among minerals were examined using bilateral Pearson correlations. Contents of mineral and protein were not independent from each other, and thus we carried out a Principal Component Analysis (PCA) using equamax rotation to reduce the variables to the minimum independent factors. The number of PCA factors was initially set to those with eigenvalue above 1 [37–38]. The scores for each factor were then used as new variables in a binary logistic regression (using a forward stepwise procedure [39]) to test for the influence of minerals and protein in plant selection (preferred or non-preferred). Plant category (herbaceous *vs*. shrubs) was included in this analysis in order to control for its possible influence in the observed preferences. All analyses were performed using SPSS (version 20.0 for Windows, IBM, USA).

## Results

Among the 35 studied plant species (belonging to 25 different families; see Table 1), we identified 23 preferred (13 herbaceous and 10 shrubs) and 12 non-preferred species (10 herbaceous and two shrubs). The mean protein and mineral composition of preferred and non-preferred plants is shown in Table 2. Pooling both plant types (herbaceous and shrubs), preferred plants showed significantly lower content in S, Cu, Sr, Zn, protein and ash. Surprisingly, only Na and P were below deficiency levels in non-preferred plants, while Na, P, S and Zn were below deficiency levels in preferred ones. Only sulphur was above tolerance levels in non-preferred plants. Table 2 also shows differences in mean composition for each plant category: preferred shrubs showed a lower S and protein content, while preferred herbaceous species had lower S, B, Sr and Zn. Na and P were below deficiency levels in non-preferred shrubs, while K, Mg, Na, P and Zn were below deficiency levels in preferred ones. Na and P were below deficiency levels in both preferred and non-preferred herbaceous plants, while S was above toxic levels in non-preferred plants.

**Table 2:** Differences in mean values (±SE) of mineral, crude protein and ash content in each plant category (herbaceous and shrubs/trees).

	**35 STUDIED PLANTS**			**SHRUBS/TREES**			**HERBACEOUS**			**Deficiency ^b^**	**Maximum Tolerance ^b^**
	**Non-Preferred**	**Preferred**	**Sig. ^a^**	**Non-Preferred**	**Preferred**	**Sig. ^a^**	**Non-Preferred**	**Preferred**	**Sig. ^a^**		
Ca (%)	1.089 ± 0.230	0.758 ± 0.118	0.163	0.59 ± 0.04	0.59 ± 0.12	0.989	1.19 ± 0.27	0.89 ± 0.18	0.345	0.3	2
K (%)	1.458 ± 0.255	1.016 ± 0.212	0.211	0.70 ± 0.11	**0.58 ± 0.11**	0.665	1.61 ± 0.28	1.35 ± 0.34	0.578	0.6	3
Mg (%)	0.255 ± 0.037	0.189 ± 0.043	0.319	0.18 ± 0.01	**0.14 ± 0.02**	0.370	0.27 ± 0.04	0.23 ± 0.07	0.671	0.15	5
Na (%)	**0.052 ± 0.024**	**0.018 ± 0.008**	0.112	**0.005 ± 0.005**	**0.003 ± 0.002**	0.624	**0.061 ± 0.028**	**0.030 ± 0.014**	0.296	0.06	0.35
P (%)	**0.177 ± 0.024**	**0.134 ± 0.018**	0.166	**0.055 ± 0.005**	**0.080 ± 0.014**	0.465	**0.201 ± 0.022**	**0.175 ± 0.024**	0.451	0.25	0.6
S (%)	*0.204 ± 0.053*	**0.092 ± 0.011**	0.009 **	0.107 ± 0.027	0.071 ± 0.006	0.058 †	*0.224 ± 0.062*	0.107 ± 0.018	0.057 †	0.1	0.2
B (mg/Kg)	28.4 ± 4.4	20.3 ± 3.0	0.132	30.6 ± 7.2	26.6 ± 5.8	0.779	28.0 ± 5.2	15.4 ± 2.4	0.028 *	^c^	^c^
Cu (mg/Kg)	7.7 ± 1.2	5.5 ± 0.45	0.048 *	7.1 ± 2.5	5.2 ± 0.5	0.236	7.8 ± 1.4	5.8 ± 0.7	0.178	4	20
Fe (mg/Kg)	250. 5 ± 53.2	156.3 ± 51.2	0.252	133 ± 38	105 ± 19	0.550	274 ± 61	196 ± 89	0.507	30	500
Mn (mg/Kg)	110.3 ± 26.7	76.2 ± 12.9	0.203	153 ± 28	99 ± 26	0.395	102 ± 31	59 ± 10	0.159	20	1000
Se (mg/Kg)	4.3 ± 0.487	4.2 ± 0.365	0.830	4.75 ± 1.05	4.03 ± 0.57	0.612	4.25 ± 0.56	4.33 ± 0.49	0.915	0.06	4–5
Sr (mg/Kg)	91.7 ± 25.4	44.2 ± 5.0	0.020 *	41.6 ± 3.4	37.4 ± 7.3	0.806	101.7 ± 29.7	49.4 ± 6.7	0.066 †	^c^	150 ^d^
Zn (mg/Kg)	34.8 ± 3.8	**24.9 ± 2.4**	0.028 *	31.3 ± 12.2	**23.0 ± 4.0**	0.430	35.5 ± 4.2	26.4 ± 3.0	0.086 †	25	750
Protein (%)	13.6 ± 2.1	9.1 ± 0.92	0.027 *	12.6 ± 5.2	6.9 ± 0.4	0.019 *	13.8 ± 2.4	10.8 ± 1.4	0.262		
Ash (%)	10.5 ± 1.8	7.1 ± 0.95	0.077 †	6.00 ± 0.60	5.26 ± 0.66	0.642	11.44 ± 2.09	8.57 ± 1.55	0.272		

Bold values are below the deficiency level, and values in italics are above the tolerance limits (only sulphur).

^a^ Significant differences between mean values at 0.1, 0.05, and 0.01 are indicated by †, * and ** respectively.

^b^ Based on [19,35].

^c^ Unknown deficiency levels.

^d^ Maximum tolerance is only known for rats [54].

**Figure 1:**
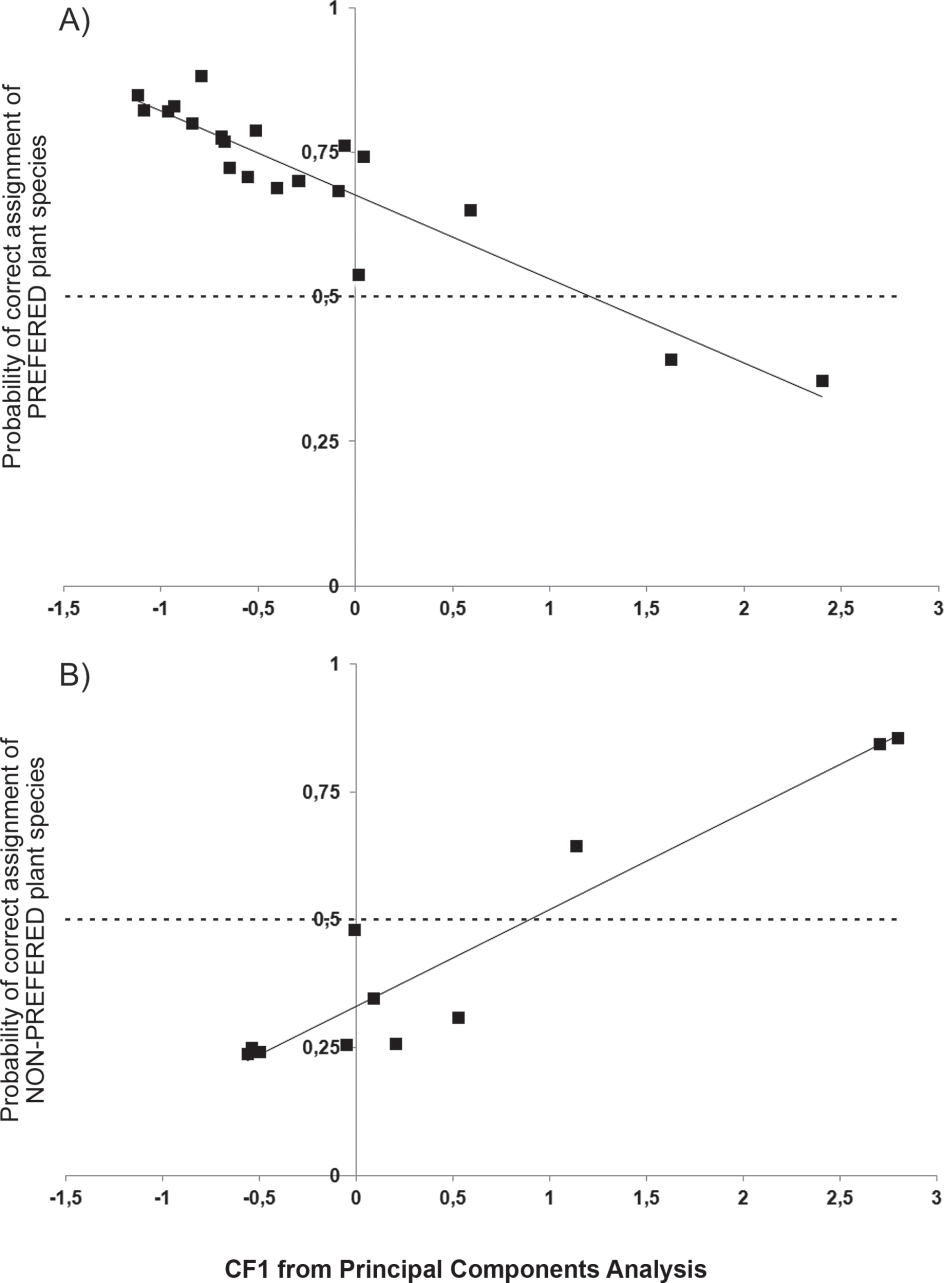
Probability of correct assignment of preferred (A) and non-preferred plans (B) in the model obtained through binary regression (see text). CF1, related to high content of K, Ca, protein, S, Sr and Zn was the only significant factor in the model (with a negative coefficient). CF2, CF3 and plant category (shrubs *vs*. herbaceous) were not significant in the model. The model was quite effective for identifying preferred plants, but poor for identifying non-preferred ones.

Table 3 shows correlations among minerals in plants. Most minerals significantly correlated among them, but also with ash and protein (except Mn which correlated only with Sr, and Se which did not correlate with any other mineral). In most cases the correlation was highly significant. Thus, protein and mineral content were reduced to 3 significant factors (accounting for 67% of the variance; Table 4) through Principal Component Analysis. The first factor (CF1) was related to the content in K, Ca, protein, S, Sr, Mg and Zn (ordered by factor loading). The second factor (CF2) was related to the content of Na. The third factor (CF3) was related to the content of Se. A binary logistic regression model showed that deer preferred plants negatively related to PCA factor 1 [Model: Coefficient for CF1 = −0.952, p = 0.030; Coefficient for constant = 0.700, P = 0.075; with absence of correlation between CF1 and constant (−0.023); not significant variables: CF2, CF3 and ‘plant category’; *Nagelkerke’s R^2^* = 0.221, Hosmer and Lemeshow goodness-of-fit test of the null hypothesis: *χ^2^* = 7.375, df = 7, P = 0.393 (the model does not adequately fit the data when P < 0.05)]. Thus, the model showed that preference decreased when CF1 increased, which means that the lower the content of K, Ca, protein, S, Sr, Mg and Zn, the more likely that a plant is preferred. This model classified correctly 71.4% of the studied plant species: 91.3% of the preferred species but only a 33.3% of non-preferred ones. Moreover, the probability of correct assignment with this model was above 60% in 20 of 23 preferred plant species, but only in 3 of 12 non-preferred plant species (Fig. 1). That means that our model based on CF1 was greatly efficient for identifying preferred plants, but not for identifying non-preferred plants.

**Table 3:** Pearson’s correlations among mineral, ash and protein content in plants in LM game estate.

	**K**	**Mg**	**Na**	**P**	**B**	**Cu**	**Fe**	**Mn**	**S**	**Se**	**Sr**	**Zn**	**Ash**	**Protein**
**Ca**	0.575**	0.610**	-	0.472**	0.544**	-	0.700**	-	0.611**	-	0.856**	0.422*	0.814**	0.567**
**K**		0.838**	0.478**	0.674**	-	0.494**	0.455**	-	0.591**	-	0.493**	0.555**	0.883**	0.675**
**Mg**			0.400*	0.490**	-	-	-	-	0.387*	-	0.530**	0.505**	0.779**	0.572**
**Na**				0.504**	0.594**	-	-	-	-	-	-	-	0.375*	-
**P**					-	-	0.399*	-	-	-	-	-	0.562**	-
**B**						-	-	-	-	-	0.461**	-	-	-
**Cu**							-	-	0.556**	-	-	0.554**	0.528**	0.556**
**Fe**								-	0.587**	-	0.509**	-	0.652**	-
**Mn**									-	-	0.456**	-	-	-
**S**										-	0.751**	0.629**	0.732**	0.759**
**Se**											-	-	-	-
**Sr**												0.563**	0.765**	0.665**
**Zn**													0.578**	0.642**
**Ash**														0.665**

Dashes indicate coefficients that were not significant.

Probability at 0.05 and 0.01 is indicated, respectively, by *, and **.

**Table 4:** Factor loadings from the Principal Component Analysis performed on protein and mineral content of the 35 plant species analyzed. The table shows the correlation between each variable and each factor. Minerals with greatest influence on the extracted factors are shown in bold (loading higher than 0.7, following [38]).

	**CF1**	**CF2**	**CF3**
Cumulated Explained Variance (%)	42.7	57.0	67.0
Eigenvalue	5.98	1.99	1.40
Ca	**0.832**	−0.294	−0.266
K	**0.837**	0.345	−0.094
Mg	**0.752**	0.199	−0.060
Na	0.417	**0.700**	0.023
P	0.564	0.507	−0.397
B	0.368	−0.465	−0.291
Cu	0.621	0.319	0.248
Fe	0.641	−0.229	−0.384
Mn	0.258	−0.594	0.397
S	**0.816**	−0.160	0.091
Se	0.061	0.213	**0.713**
Sr	**0.814**	−0.442	0.088
Zn	**0.723**	−0.093	0.324
Protein	**0.823**	0.056	0.284

## Discussion

Our study shows that the plant species preferred by red deer in Mediterranean ecosystems have lower contents in protein and minerals (S, Cu, Sr, Zn) than the non-preferred plants. By plant categories, preferred herbaceous species had lower S, B, Sr and Zn, and preferred shrubby species had lower S and protein. Most of the studied minerals and the protein were highly and positively correlated, suggesting that plants which are rich in some minerals are rich in most of the others, and highlighting the difficulty to untangle the effect of each mineral on dietary selection studies [1]. However, a factor explained by protein and mineral content showed a great capability to classify preferred plants, since 91.3% of them (21 of 23) were correctly assigned. In contrast, its capability to identify non-preferred plant species was low (only 33.3% of correct assignments; 3 of 11). This highlights the great importance of minerals and protein to identify preferred plant species by red deer, but also suggests a much more complex pattern in diet avoidance. Indeed, the preference for a plant species has not been previously associated to a single nutrient but rather to a combined effect of nutrients and defence compounds [40–42]. It is probable that most of the non-preferred plant species in our study site contain highly irritant principles, typically high in evergreen sclerophyllous Mediterranean shrubs [43–44]. In fact, tannin and lignin concentrations are actually relatively high in the vegetation in this area, but this was likely not related to rejection as red deer select here plants with high content in tannins [32]. This is not surprising because deer have tannin binding salivary proteins [36], and thus, it seems that high concentrations of irritant compounds in plants are not a determinant factor affecting diet selection. Anyway, some of the observed non-preferred herbaceous plants and one of the shrubs (*Daphne* genus) have been described as rich in other secondary plant compounds (*e.g.*, pyrrolizidine alkaloids, hypericin, soluble oxalates, tropane alkaloids, coumarin glycosides) that are linked to a number of toxic related effects to ruminants [45,46], which combined with the contents of the analysed nutritional compounds could well explain their avoidance by red deer.

Preference for plants with lower protein and mineral content seems a counterintuitive result. However, after a detailed examination of the results these are still surprisingly coherent with OFT. Our results show that the content of protein in preferred plants was 9.1% in average (lower than in non-preferred ones; 13.6%). Although knowledge of diet protein requirements for cervids is limited and widely variable [47], values of preferred plants coincide with the recommendation made by [34] for maintenance of cervids, which mentioned that the minimum requirements are 8 to 10% (depending on the season). Requirements may increase up to 15% during lactation and antler growth [34]. Nevertheless, we should not forget that these recommendations are usually based on extrapolation from livestock under highly productive systems. In fact, [48] recently showed that 8% of dietary protein is enough for lactating hinds if energy content is also adequate. Similarly, 12 *vs*. 16% dietary protein level had no statistically significant effect on velvet antler yield in sika deer (*C. nippon*) [49]. Other studies also failed to find this dietary protein effect in antler growth in red deer [50], white-tailed deer (*O. virginianus* [51]), and sika deer [52]. This result also agrees with the selection for low protein content compared to diets predicted through random simulations (considering availability of species and selectivity) calculated for red deer by [42]. In contrast, [14] found a selection of plants with greater protein content (9 *vs*. 7%), but this pattern was observed in a suboptimal Mediterranean habitat where the protein content in non-preferred plants was clearly lower than the minimum requirements. Similarly, [53] also failed to find an influence of protein in diet selection by white-tailed deer when it was supplied in adequate minimum threshold (12%). Thus, our studied population seems to be selecting for plants with an overall adequate level of protein either for maintenance and productive periods. This agrees with the main principle of OFT which states that herbivores first satisfy their needs on the most important nutrients (energy and protein) and thereafter imbalance the ingestion of other nutrients. And according to our results, it seems that it is at this point when minerals play their role.

When mineral contents in the studied plant species are compared to requirements, minimum thresholds and toxicity levels for cervids (Table 2; extracted from [19,35,54]), we can observe that Ca, B, Cu, Fe, Mn, Se, Sr and Zn are adequate both in preferred and non-preferred plant species. Therefore, it is unlikely that preference or avoidance of the studied plant species could be actually driven by these minerals. The logistic regression model shows a negative effect of CF1 on diet preferences, which means that preference is influenced by a lower content of K, Ca, S, Sr, Mg, and Zn. Discarding those minerals which are found in adequate amounts (previously discussed; Table 2), only K, Mg and S may have some actual effect on the observed pattern of plant selection. However, deficiencies of K and Mg in preferred shrubs are exiguous, and ANOVAs show that there are no statistical differences between K and Mg contents in preferred and non-preferred plants, but even a tendency for greater content in non-preferred ones. However, sulphur is well supported by our results as key mineral: 1) it appears in CF1 with a high score; 2) it also has the greatest difference in content between preferred and non-preferred plants (lowest P-value; Table 2); 3) it is the only mineral showing consistent differences between preferred and non-preferred plants both in the ANOVA analyses for herbaceous and shrubby species; but especially 4) it is the only mineral reaching toxic levels in non-preferred plants, both when pooling all the studied plants and when analysing only herbaceous plants.

Early studies in ruminants suggested adequate requirements for S around 0.4% [55], but further experiments showed negative effects with concentrations above 0.2% [20]. Our results show that non-preferred plants are slightly above the toxic level of S (0.2%, Table 2) while preferred plants are in a safe level (0.1%). This negative effect of S has also recently been supported by [22] who showed a linear decrease in average daily gains and dry matter intake when increasing dietary sulphur from 0.12 to 0.46%. The consumption of the studied non preferred plants may not only lead to symptoms of intoxication by S, but also to secondary deficiencies of Cu and Se (as high concentrations of S reduce their absorption [19]). This indirect effect of S has been just once reported in free-ranging herbivores [56]. Recent studies in livestock have also shown lower concentrations of Cu [22,57] and Se [58] in plasma when dietary sulphur was over 0.2%, and these sulphur-induced deficiencies may lead to severe diseases [21]. Both effects (direct negative effects of high dietary sulphur and indirect effect through reduction of Cu levels) have been recently proved in cattle through meta-analysis [23]. Nevertheless, our study highlights for the first time that these negative effects of sulphur may drive diet preferences in large herbivores in the wild. In conclusion, minerals and protein content in preferred *vs*. non-preferred plant species in Southern Spain suggests that low sulphur content may be a key factor explaining the preference for certain plant species.

Our results provide new insights on the overall annual patterns linking deer plant selection and a wide range of nutritional contents of plants. However, strong seasonal variations on vegetation availability and nutritional contents are characteristic of the Mediterranean region where summer drought leads to particularly low nutritional plant contents and lignification towards the end of the dry hot summer (September) with increases in plant quantity and quality in the rainy spring (April-June) and autumn (October-November). This high temporal heterogeneity in nutritional supply together with deer phenology would likely result in complex selectivity processes for minerals by deer across seasons. Additionally the observed effect of high S contents on plant avoidance by red deer could interact with the toxic effects of multiple secondary plant compounds leading to an increased toxicity of species containing both. These synergetic effects have not been documented for S, but for other minerals or among different secondary compounds [9,59–60]. Further research accounting for the intricate relationships between the vegetal and animal components of dietary selection could substantially build on the outcomes of the present study and help to improve our understanding on how mineral and protein contents drive use of resources by deer. In this regards, further “Cafeteria feeding trials” are necessary to confirm our results in settings including quantitative preference indexes [61–62].

## Supporting Information

S1 Dataset The dataset used in this manuscript. (XLSX)
